# Targeting metabolism in aortic aneurysm and dissection: from basic research to clinical applications

**DOI:** 10.7150/ijbs.85467

**Published:** 2023-07-31

**Authors:** Qi Wang, Gulinazi Yesitayi, Bingyan Liu, Dilixiati Siti, Mierxiati Ainiwan, Aliya Aizitiaili, Xiang Ma

**Affiliations:** 1 Department of Cardiology, The First Affiliated Hospital of Xinjiang Medical University, Xinjiang Medical University, Urumqi, China.; 2 Environment Research Institute, Shandong University, Qingdao 266237, China.

**Keywords:** Metabolites, Aortic aneurysm and dissection, Gut microbes, TCA cycle, Clinical applications.

## Abstract

Aortic aneurysm and dissection (AAD) are a group of insidious and lethal cardiovascular diseases that characterized by seriously threatening the life and health of people, but lack effective nonsurgical interventions. Alterations in metabolites are increasingly recognized as universal features of AAD because metabolic abnormalities have been identified not only in arterial tissue but also in blood and vascular cells from both patients and animal models with this disease. Over the past few decades, studies have further supported this notion by linking AAD to various types of metabolites such as those derived from gut microbiota or involved in TCA cycle or lipid metabolism. Many of these altered metabolites may contribute to the pathogenesis of AAD. This review aims to illustrate the close association between body metabolism and the occurrence and development of AAD, as well as summarize the significance of metabolites correlated with the pathological process of AAD. This provides valuable insight for developing new therapeutic agents for AAD. Therefore, we present a brief overview of metabolism in AAD biology, including signaling pathways involved in these processes and current clinical studies targeting AAD metabolisms. It is necessary to understand the metabolic mechanisms underlying AAD to provides significant knowledge for AAD diagnosis and new therapeutics for treatment.

## Introduction

Aortic disease is a large group of vascular diseases with high mortality rates, in addition to coronary heart disease (CHD) and peripheral artery disease^1^. The incidence of primary aortic diseases, including aortic aneurysm and dissection (AAD), increased from 2.49/100,000 to 2.78/100,000 between 1990 and 2010, with higher mortality rates in older individuals and males^2^. Excluding underlying genetic alterations, sporadic AAD is associated with smoking, hypertension, old age, and male gender^3^. Mechanically, the pathogenesis of AAD involves vascular smooth muscle cell (VSMC) phenotype switch, endothelial dysfunction, inflammation, cells apoptosis, and degradation of the extracellular matrix (ECM)^4^. D-dimer is of great significance in the diagnosis of AAD, which is widely used in clinical practice for AAD risk assessment with their increase suggesting the presence of AAD^5^. Unfortunately, classical biomarkers can only partially explain the distribution of AAD risk in the general population and numerous metabolic drivers of the AAD risk are underestimated. Additionally, while computed tomography angiography remains the clinical standard for AAD diagnosis and monitoring, they are less suitable for mass screening and large-scale trials^6^.

Although drug therapy, such as β-adrenergic receptor antagonists and angiotensin receptor type I antagonists, can alleviate the enlargement of aneurysms, no effective medication has been found to prevent the occurrence and progression development of AAD in clinical practice^7^. This indicates that the current lack of accurate biomarkers and effective targets for the diagnosis and therapy limits the prevention and treatment of AAD^8^. It is therefore necessary to further explore the potential molecular targets of AAD in order to develop more effective early prevention strategies, thereby reducing the associated morbidity and mortality.

To date, numerous studies have illustrated that metabolites that are involved in vascular injury and repair by interacting with AAD risk factors are the most prominent factors driving AAD. ​In this context, the potential for reducing AAD mortality and improving prognosis through the use of metabolic therapy is great. Although an increasing number of metabolomics studies, an emerging analysis technique, have linked AAD to different low-molecular-weight-metabolites (LMWMs) 9, 10, there is still a lack of a comprehensive understanding of the metabolic profile of AAD has hampered our understanding of the biology of the hallmark metabolites and their role in AAD pathogenesis. ​A deeper understanding of the essential function of metabolites in the pathophysiology of AAD may remain crucial for achieving significant breakthroughs in the diagnosis and treatment of AAD. This review summarizes recent advances in this field and aims to provide a review of the existing literature on the relationship between metabolites and AAD, thereby discussing their regulatory role in the development of AAD as well as the current status and future outlook on their potential as diagnostic markers and therapeutic targets.

## Metabolites as candidate biomarkers of AAD

**Figure 1:**
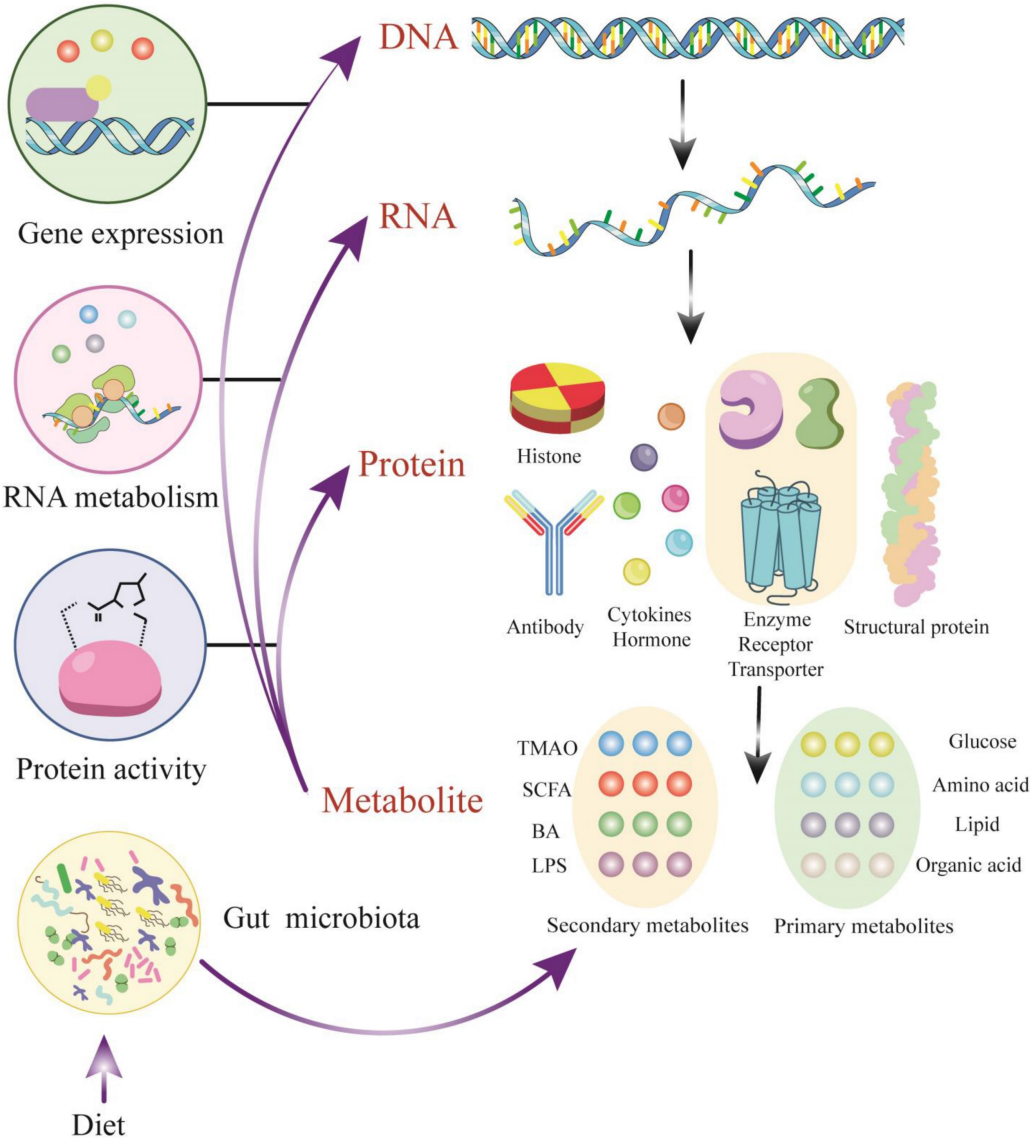
** Metabolites - active modulators of gene and protein activity.** DNA transcription and RNA translation yield proteins with a broad range of structure and function. A subset of proteins including enzymes and transporters plays a key role in modulating metabolites, which are derived from endogenous metabolism and exogenous sources such as diet and the microbiome. Metabolites could actively regulate protein activity, serve as signaling molecules for transcription factors and be involved in epigenetic regulation. Abbreviations: TMAO, trimethylamine N-oxide; SCFA, short-chain fatty acid; BA, bile acid; LPS, lipopolysaccharide; DNA, Deoxyribonucleic Acid; RNA, Reibonucleic Acid.

Metabolomics is a burgeoning field in the life sciences, the emergence of which is inevitable for life science research^11^. It utilizes a combination of advanced techniques in analytical chemistry and comprehensive statistical methods to fully characterize the metabolome, focusing on the qualitative and quantitative analysis of LMWMs with a relative molecular weight of less than 1500 in a certain organism or cell^12^. Metabolites are the ultimate products of gene expression, generated by the action of metabolic enzymes. They reflect variations in the genome, transcriptome, and proteome and indicates the endpoint of the 'omics' cascade. The metabolome interacts with and actively modulates all other 'omics' levels (Figure 1). Although metabolites are small molecules compared to genes or proteins, the importance of metabolites that should not be underestimated is due to the fact that they are commonly present in a given organ elle, cell, organ, biofluid or organism rather than dead cells^13^.

LMWMs in the metabolome contain both endogenous sources from genetics and exogenous sources which mainly including nutrition, drugs, and environment. Endogenous metabolites are highly conserved and represent the collections of similar LMWMs, albeit at different concentrations, in different species^14^. However, exogenous metabolites are highly variable and mainly reflect dietary and environmental factors, as well as the composition of the microbiome. Both endogenous and exogenous LMWMs fulfill an essential function in normal and pathological processes^15^. Given that the metabolome is composed of thousands of individual chemicals attaching to diverse chemical classes^16^, more extensive and precise equipment is needed to ensure the accuracy of metabolome measurements compared with genomics or proteomics. Nuclear magnetic resonance (NMR) spectroscopy and mass spectrometry (MS), typically coupled to gas chromatography-mass spectrometry (GC-MS) or liquid chromatography-mass spectrometry (LC-MS), as well as capillary electrophoresis-mass spectrometry (CE-MS), and ion mobility spectrometrymass spectrometry (IMS-MS), are the most commonly used detection and analysis techniques^17^.

Recent studies have shown that metabolites do not only maintain normal physiological functions of living organisms, they can serve as regulators of pathological processes in diseases such as cardiovascular disease (CVD)^18^. Endogenous metabolites, also known as primary metabolites, are encoded by the host genome and they are responsible for the growth, development, and crucial physiological functions of the body^19^. On the other hand, secondary metabolites are often referred to as exogenous metabolites from diet or environment that may not be essential for growth and development or even harmful^20^, ^21^. Endogenous metabolites and exogenous metabolites play important roles in the pathogenesis of AAD and have potential as new biomarkers for diagnosis, gaining popularity in various areas of medical research. Elevated plasma succinate levels were shown to reliably differentiate patients with AAD from those with acute myocardial infarction (AMI) or pulmonary embolism (PE) when presenting with chest pain, suggesting that succinate could be used as a biomarker for pre-diagnosis^9^. Elevated levels of trimethylamine N-oxide (TMAO) were found to be associated with the incidence and growth of abdominal aortic aneurysms (AAA) in two case-control cohorts. Furthermore, there was a significant correlation between higher TMAO concentrations and abdominal aortic diameter^22^. Additionally, impaired metabolism of aromatic microbial metabolites (AMMs), as detected by levels in the blood before and after surgery, was found to be associated with postoperative complications in AA patients^23^. In summary, metabolomics analysis appears to improve the risk stratification and early identification of AAD, while also holding promises for enhancing our understanding of the pathophysiological processes that lead to AAD.

## Gut microbiota-derived metabolite implications in AAD

### Characteristics of the gut microbiota

The term 'microbiota', a hidden endocrine organ, contributes to 150 times more genetic information than the entire human genome, which could date back to early 1900s^24^. Various parts of the human body coexist with a large number of microbes composed of bacteria, yeasts, and viruses in the gut, skin, mouth and lungs. Generally, the gastrointestinal tract is considered to host the largest and most variable number of microbes^25^. It is widely acknowledged that gut microbiota is the most essential microbiota for maintaining our health, which is comprised of six phyla (Firmicutes, Bacteroidetes, Actinobacteria, Proteobacteria, Fusobacteria, and Verrucomicrobia) 26. Among them, Firmicutes and Bacteroidetes make up the vast majority of healthy intestinal environments in adults. The Firmicutes/ Bacteroidetes ratio is defined as a signature indicator that leads to exert healthy intestinal microbiota^27^. Meanwhile, gut bacteria play important roles in food fermentation, pathogen resistance, immune response stimulation, and vitamin production.

### Gut microbiota dysbiosis and AAD

In a healthy state, the human intestine barrier serves as a defense to protect against the translocation of microorganisms or microbial products into the bloodstream. It has been maintained by the tight junctions of epithelial cells, mucus production, mucosal immunity, and the gut-vascular barrier^28^. In AAD patients, it has been observed that the serum levels of diamine oxidase (DAO) reflecting impaired gut barrier function at an early stage were significantly higher 29. The presence of microbiota dysbiosis can lead to leaky intestines. For example, LPS from most Gram-negative bacteria increase intestinal permeability^30^. Microbiota dysbiosis refers to an unbalanced interaction between host and microbe, which is mainly manifested in two aspects including altered composition of gut microbiota, and gut microbiota-derived signaling molecules. Ultimately, this leads to the pathophysiological diseases such as CVDs including atherosclerosis, myocardial infarction (MI), hypertension, and atrial fibrillation (AF)^31^-^33^. According to previous studies, only a few have investigated the changes in gut microbiota between patients with AAD and healthy participants. Gut microbiota diversity did not differ significantly between patients with AAD and atherosclerosis, however, AAD patients had higher relative abundance of Leuconostocaceae and Faecalibacterium while lower levels of Firmicuteria and Selenomonadales^34^. A recent study revealed significant changes in the composition of gut microbiota in Ang II-induced AAA mice^35^. Akkermansia was reduced in AAA mice, and Odoribacter, Helicobacter and Ruminococcus were significantly positively correlated with the abdominal aortic diamete, indicating their potential role in the progression of AAA 35. Tian et al. revealed gut microbiota dysbiosis in AAA patients, which was associated with the occurrence of AAA^36^. Surprisingly, R. intestinalis alleviated AAA by inhibiting neutrophil infiltration and phenotypic switching of VSMCs.

Consequently, the constituent species of the intestinal flora and their relative abundance are factors that may modify susceptibility to the development of AAD. Although there are few studies clarifying the specific underlying mechanism of intestinal flora changes affecting AAD, which cannot fully clarify the crucial cause-effect relationships, it still suggests the potential of gut microbiota as therapeutic applications target for AAD.

### The crosstalk of dietary habits and metabolites in the development of AAD

Diet plays a crucial role in shaping the compositional and functional characteristics of gut microbiota in both humans and animals. Various studies have shown that the absorption of nutrients from food is influenced in part by the composition of an individual's gut microbiota, which in turn is shaped by food^37^. Caloric restriction (CR), which involves a long-term reduction in total caloric intake without malnutrition, is the only known strategy to reliably improve health and longevity in most living organisms^38^. Meanwhile, CR has been reported as a nonpharmacological intervention to prevent AAA formation^39^. Gao et al. suggested that CR reduces the occurrence of AA by maintaining p53-mediated mitochondrial function. However, in the absence of p53, CR inversely promoted AAA formation accompanied by smooth muscle cell mitochondrial dysfunction^40^. Moreover, the ketogenic diet significantly reduced the incidence of AAA expansion and rupture in mice, accompanied by a significant reduction in inflammatory cytokine content and infiltrated macrophages in AAA tissue^41^. In addition, a retrospective cross-sectional cohort study in the United States revealed a correlation between fruit and vegetable intake and AAA risk^42^. Similarly, a prospective cohort study carried out in Sweden found that only fruit intake was associated with AAA risk^43^. Furthermore, another prospective Swedish cohort study emphasized the importance of fibre and plant foods for reducing AAA risk. High consumption of fruits, berries, and vegetables, particularly leaf vegetables, were linked to decreased AAA incidence^44^. Conversely, a high-fat diet increases AAA diameter significantly and the risk of rupture by accelerating chronic inflammation^45^. In conclusion, recent studies are beginning to reveal the biological mechanisms that mediate the beneficial effects of healthy dietary patterns of AAD. However, studies on the role of dietary habits and metabolites in the development of AAD are still in the preliminary stages and more mechanistic studies are needed to understand the interactions between dietary habits, microbiome and metabolites in the key molecular pathways regulating AAD.

### Microbiota-Derived Metabolites and AAD

**Figure 2:**
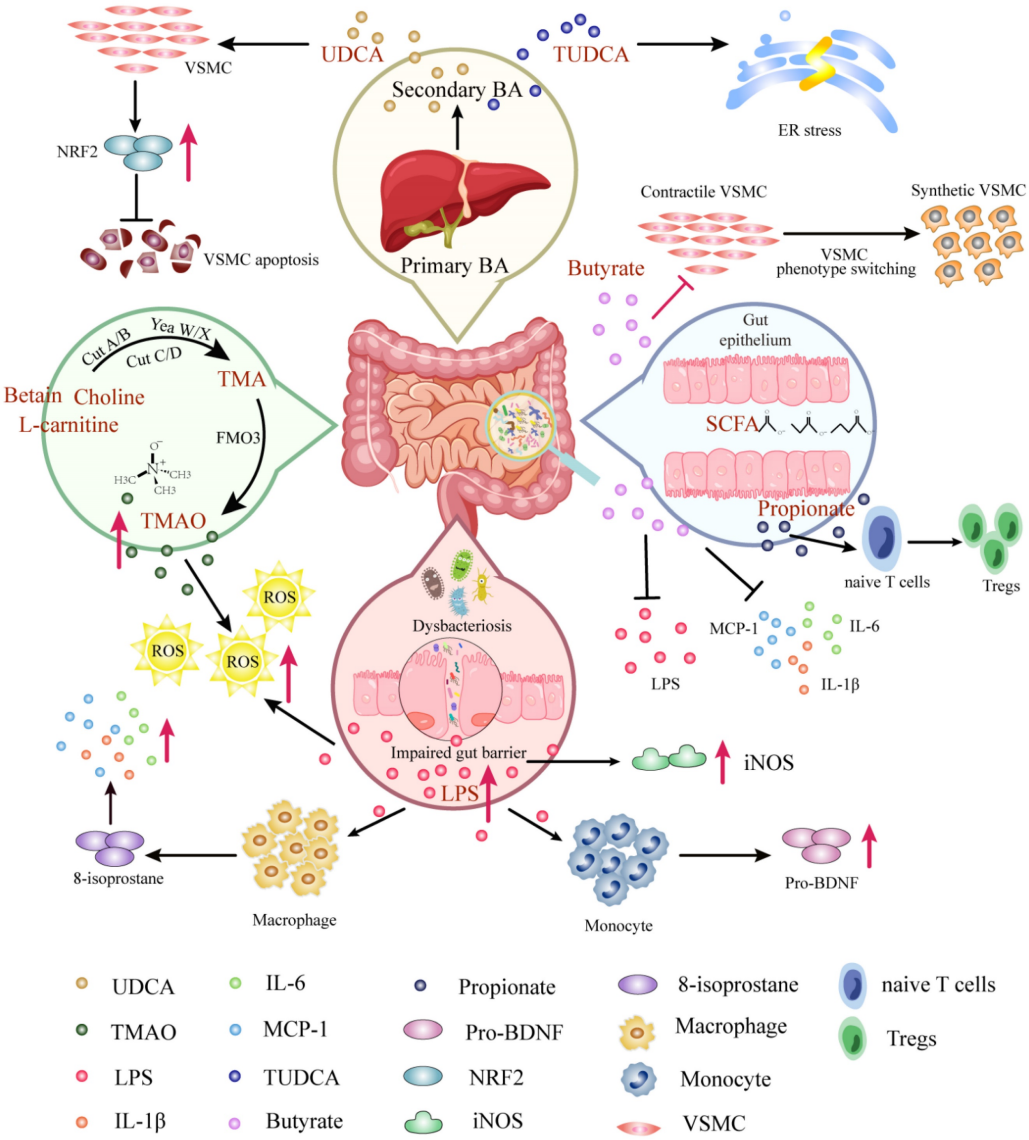
** Mechanisms of gut microbiota-derived metabolites in AAD.** Molecular pathways and host receptors that link gut microbiota-derived metabolites with AAD and cellular phenotypes, mainly including TMAO, SCFA, BA, and LPS. Abbreviations: VSMC, vascular smooth muscle cell; UDCA, ursodeoxycholic acid; TUDCA, tauroursodeoxycholic acid; BA, bile acid; SCFA, short-chain fatty acid; ER, endoplasmic reticulum; NRF2, nuclear factor erythroid 2; TMA, trimethylamine; TMAO, trimethylamine N-oxide; FMO3, flavin lpscontaining monooxygenase 3; LPS, lipopolysaccharide; ROS, reactive oxygen species; IL-6, interleukin-6; IL-1β, interleukin-1β; iNOS, ; pro-BDNF, brain-derived neurotrophic factor precursor.

The gut microbiota participate in the uptake of nutrition and energy, and producing multifarious metabolites to maintain physiological function. There are a number of microbiota-derived metabolites, including short-chain fatty acid (SCFA), bile acid (BA), TMAO, amino acid derivatives, vitamins, lipopolysaccharide (LPS), antioxidants, and hormones in the gut^46^. Microbiota-derived metabolites have been demonstrated to be able to penetrate through the gut mucosa layer into the bloodstream to migrate to distant organs, leading to exert synergistic effects in healthy individuals and have significant impacts on the regulation of CVD^47^. The first research revealing the possible causality between the microbiota-derived metabolite and CVD focused on TMAO, derived from L-carnitine, promotes atherosclerosis.^48^ Emerging evidence supports that microbiota-derived metabolites, discussed below, are correlated with the occurrence and recovery of AAD (Figure 2).

The intestinal flora produces trimethylamine (TMA) from metabolism of choline, lecithin and L-carnitine by the fermentation of dairy products such as eggs, fish and meat. Then TMA is converted to TMAO via the flavin-containing monooxygenase 3 (FMO3) enzyme in the liver^49^. Undoubtedly, TMAO is the most distinguished gut microbiota-derived metabolite which leads to AAD. It was found that TMAO levels were significantly higher in AAD patients compared to healthy people, while their precursor carnitine, choline and betaine levels were significantly lower^10^. Consistently, experimental studies proposed that TMAO promoted AAA formation by inducing ROS accumulation and aggravating aortic smooth muscle cell senescence in mice^50^. Overall, the existing researches showed a dose-positive correlation between TMAO concentration and the occurrence of AAD, which was directly involved in the pathogenesis of AAD. However, detailed and mechanism investigations are needed to understand the interaction between TMAO production and AAD risks.

Gut microbiota participates in bile acid (BA) metabolism, generating associated unconjugated and secondary BAs^51^. A key contribution of BAs has been proved to facilitate emulsifying and absorbing fat-soluble dietary nutrients. The primary BAs (e.g., chenodeoxycholic acid) are modified via intestinal flora and bile salt hydrolase in the ileum, then they will be converted by BA 7α dehydrogenation, dihydroxyacetone, or authorization to yield secondary BAs (e.g., ursodeoxycholic acid), numerous of which serve as hormone-like roles^52^. More recent studies have shown some structurally specific BAs play additional crucial part, including but not limited in regulating AAD. Ursodeoxycholic Acid (UDCA) has been shown to inhibit oxidative stress by up-regulating nuclear factor erythroid 2 (Nrf2) expression in VSMCs, which contribute to the decrease of VSMCs apoptosis, leading to suppress acute aortic dissection formation^53^. Alternatively, the tauroursodeoxycholic acid (TUDCA) formed by condensation between the carboxyl group of UDCA and the amino group of taurine, palys a role in attenuating Angiotensin II induced AAD formation by restraining endoplasmic reticulum stress^54^. It has been suggested that UDCA and TUDCA have clinical potential to be novel targets for AAD therapy.

LPS is an endotoxin originating from Gram-negative bacteria, especially Escherichia genera^55^. When the gut barrier is impaired, LPS can enter the host circulation where recognized by toll-like receptors (TLRs) on the cells surface, inducing the release of proinflammatory cytokines to result in a proinflammatory host state^56^. While the role of LPS directly on the pathogenesis of AAD has not been investigated, antibodies against Porphyromonas gingivalis (Pg) and Aggregatibacter actinomycetemcomitans (Aa), LPS, and elevated CRP levels are present in all AAA patients. Notably, the presence of Pg in saliva correlated significantly with LPS levels in blood^57^. Indirectly, a suppressed cytokine response was observed in monocyte-derived macrophages treated with LPS in the plasma of AAA patients via reducing 8-isoprostane. The evidence illustrated that macrophages exhibited a recalcitrant phenotype during AAD pathology featuring inhibition of inflammatory cytokine production following LPS challenge^58^. In addition, LPS induced the monocyte supernatanta significant release of brain-derived neurotrophic factor precursor (proBDNF), which was upregulated in M2-like monocytes of AD patients^59^. Overall, LPS may contribute to an essential trigger for the activation of the inflammatory response, increasing the risk of AAD by reducing the ability to produce pro-inflammatory mediators that augment the immune response. On the other hand, LPS has been demonstrated to induce iNOS expression in arterial walls in AAA, and LPS-induced vascular inflammation fulfills an essential function in aneurysm initiation^60^.

SCFAs are produced by intestinal flora and play a role in the fermentation of resistant dietary carbohydrates, which can then enter the circulation and regulate host physiological processes^61^. SCFAs primarily consist of acetate, propionate, and butyrate, and their main receptors of action are intracellular specific G protein-coupled receptors (GPR) 41 and histone deacetylases^62^. It has been proposed that the butyrate produced by Roseburia intestinalis can ameliorate intestinal barrier function to counteract endotoxemia and provide protection against atherosclerosis^63^, suggesting that SCFAs might prevent CVD progression. In addition, SCFAs can mitigate the inflammation response induced by LPS and regulate the elevated blood pressure that is involved in AAD risk factors, suggesting that SCFAs have a theoretical protective effect against AAD. For instance, Tian et al. discovered that butyrate suppressed neutrophil infiltration and neutrophil extracellular traps formation to reduce the expression of inflammatory factors and matrix metalloproteinases (MMPs), then hinder the phenotypic switching of VSMCs in AAA^36^. Moreover, propionate plays a protective role to alleviates AAA by expanding the pool of regulatory T cells (Tregs) in the colonic lamina propria (cLP), which promotes Tregs recirculation through colonic draining lymph nodes to the inflamed aorta^64^. Nevertheless, additional investigation is needed to assess the prophylactic potential for AAD.

## Amino acid metabolism in AAD

The metabolism of amino acids has been established to be critical for AAD. Raman microspectroscopy analysis of aortic tissue from mice and humans revealed that alterations in amino acid metabolites, including phenylalanine, tyrosine, tryptophan, cysteine, aspartate, and glutamate, occur in aortic tissue from ascending thoracic aortic aneurysms (aTAA)^65^. Another study demonstrated a correlation between amino acid metabolites and AAA growth. Significant reductions in four amino acids, including histidine, aspartate, leucine, and isoleucine, were found in abdominal aortic aneurysms, but no abdominal aortic aneurysm growth rates were found^66^. Plasma amino acid profiles were markedly different in patients with AAD compared with patients with coronary artery disease. Amino acid metabolites such as histidine, glycine, serine, and citric acid differed in the AD group. Targeted metabolomic studies of angiotensin II-induced AAD in Ldlr^-/-^ mice showed that polyamines and asymmetric dimethylarginine (ADMA) were elevated in mouse aortic tissue. Furthermore, decreases in glutamine, glycine, taurine, and carnitine concentrations and increases in branched-chain amino acid (BCAA) concentrations were observed in the blood of AA patients^67^. Overall, these studies suggest that amino acid metabolites have the potential to exploit a promising new and non-invasive diagnostics for AAD.

### Homocysteine

Homocysteine (Hcy) is one of the most investigated amino acids in AAD. It is known that the only origin of Hcy in the human body is the dietary protein methionine (Met), one of the eight essential amino acids^68^. Hcy is generally not involved in the genetic code and is therefore considered a nonprotein-producing amino acid^69^, ^70^. Due to various factors such as genetic or nutritional deficiencies, Hcy cannot be metabolized to Met or Cys, which results in the accumulation of Hcy and its protein-related metabolites in the human organism, inducing a variety of pathological states^71^. Over the past years, research in the field of homocysteine has experienced incredible development, focusing on seizing the significance of homocysteine and its metabolites in CVDs.

Hyperhomocysteinemia (HHcy), elevated concentration of Hcy in the blood, has been confirmed the role in AAD. We identified 7 clinical studies aimed at shedding light on the association between HHcy and patients with AAD compared to normal participants (Table 1). We identified some common traits of these studies: (1) Serum total homocysteine (tHcy) concentrations and aortic tissue Hcy expression were abnormally elevated in the patients with AAD than in healthy controls. (2) The proportion of HHcy was significantly higher in the patients with AAD than in normal controls. (3) Hcy damages the wall of thinner abdominal aortic aneurysm segments. (4) HHcy tended to be associated with a greater risk of AAD patients. The cohort suggests that HHcy is an independent risk factor for AAD and larger prospective cohort studies are warranted to validate these findings.

**Figure 3:**
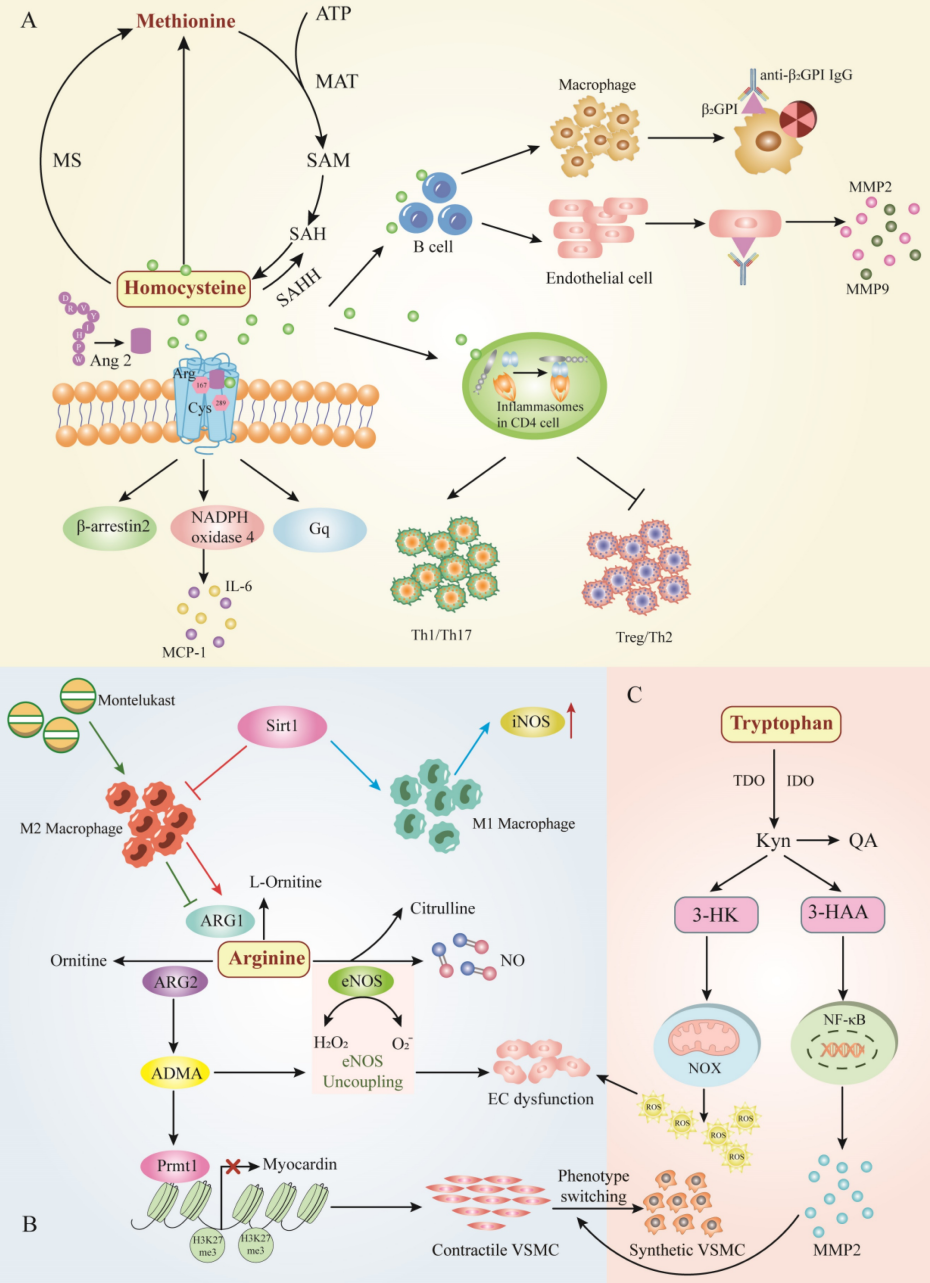
** The major amino acid metabolic pathway involves AAD.** A.Homocysteine metabolism, B. Arginine metabolism, and C. Tryptophan metabolism. Hcy exacerbates vascular inflammation by inducing immune cells such as B cells and T cells, thereby increasing the incidence of AAD. Abbreviations: ATP, adenosine triphosphate; MAT, methionine adenosyltransferase; SAM, s-adenosylmethionine; SAH, S adenosyl L homocysteine; MS, methionine synthetase; SAHH, S adeno sylhom ocysteine hydrolase; Tfh, follicular helper T cells; Th17, T helper cell 17; NADPH, nicotinamide adenine dinucleotide phosphate; β2GP1, β2-Glycoprotein 1; MMP2, matrix metalloproteinase 2; MMP9, matrix metalloproteinase 9; MCP-1, monocyte chemoattractant protein 1; IL-6, interleukin-6; Arg, arginase; Cys, cysteine; Ang 2, angiopoietin 1; iNOS, inducible nitric oxide synthase; Sirt1, Sirtuin1; ARG1, arginase 1; ARG2, arginase 2; NO, nitric oxide; ADMA, asymmetric dimethylarginine; eNOS, endothelial nitric oxide synthase; EC, endothelial cell; NOX, NAD(P)H oxidase; 3-HK, 3-hydroxykynurenine; Kyn, kynurenine; QA, quinolinic acid; 3-HAA, 3-hydroxyaminobranoic acid; TDO, tryptophan-2,3-dioxygenase; IDO, indoleamine 2, 3-dioxygenase.

Consistent with these studies, studies conducted in animal models suggested that Hcy act as a risk factor to worsen the prognosis of AAD in experimental models. Primarily, excessive Met which is the only source of Hcy exacerbates the development of AAA in Rats^72^. Hcy, serving as a partial agonist of the angiotensin II type 1 (AT1) receptor, directly induced the AT1 receptor activation via the Arg167 and Cys289 sites, which aggravated aneurysmal vascular injuries in AAA mice^73^. Similarly, Liu et al. detected that adventitial fibroblast from Apoe^-/-^ mice supplemented with excess Hcy in drinking water exaggerated inflammation due to the activation of nicotinamide adenine dinucleotide phosphate (NADPH) oxidase 4, which increased the incidence of AAA^74^. Hcy can also contribute to vascular inflammation by inducing the production of pathogenic anti-β2GPI that derived by B cell, aggravating AAA formation, the β2GPI antigen expressed on ECs formed immune complexes with anti-β2GPI antibodies and might participate in HHcy-aggravated AAA formation^75^. In addition to playing a role in AAA by participating in systemic immune imbalance, Hcy can also modulate local autoimmune processes. Hcy was investigated to cause the imbalance between follicular helper T cells (Tfh) and T helper cell 17 (Th17) via up-regulating the expression of inflammasomes which participated in humoralnfactor-induced CD4 cell imbalance, resulting in inflammasome-induced local autoimmunity in AAA^76^. Additionally, moderately elevated homocysteine does not contribute to thoracic aortic aneurysm in mice^77^. Taken together, excessive Hcy can aggravate the occurrence and development of AAA, but physical high homocysteine levels, not within the scope of wild type mice induced abnormal aortic growth. Therefore, targeting homocysteine levels is an attractive intervention goal which can be achieved by manipulating the rise of Hcy levels in physiological range to inhibit the development of AAA (Figure 3A).

### Arginine

Arginine a semi-essential amino acid that serves as a substrate for nitric oxide synthase (NOS) and arginase (ARG), the prominent NOS isoform is endothelial-type nitric oxide synthase (eNOS) in aortic vessels, which converts ARG to nitric oxide (NO)^78^. NO, acting as a vasodilator, plays an endothelial protective role in the cardiovascular system^79^. However, ARG catabolizes arginine to ornithine and urea, which affects the bioavailability of arginine and thus limits NO production in the AAD.

The main product of classically activated macrophages (M1) is NO, which requires the infusion of arginine and oxygen. The products of the inducible nitric oxide synthase (iNOS) reaction are citrulline and NO. Nevertheless, alternatively activated macrophages (M2) do not produce NO but instead utilize arginase 1 (ARG1) to hydrolyze large amounts of imported arginine^80^. Recent studies have shown that ARG1 could target the polarization of macrophages to play an essential role in the pathogenesis of AAA. Sirtuin (SIRT1)-deficiency increased iNOS induced by pro-inflammatory M1 molecules and decreased anti-inflammatory molecules produced by M2, accelerating Ang II-induced AAA formation^81^. Interestingly, montelukast significantly induced infiltration of M2 to enhance the expression of the ARG1, which suppresseed aneurysmal dilation. Consistently, increased the protein expression of ARG1 inhibited MMPs, resulting in a significant reduction in the diameter of AAA^82^. In conclusion, the balance between macrophage activation and polarization is vital for AAD pathogenesis. Targeting this balance, arginine might represent a new prognostic or therapeutic target in aneurysmal disease.

Arginine is synthetic from citrulline via the cytosolic enzymes argininosuccinate synthase (ASS) and argininosuccinate lyase (ASL). Citrulline can be produced from a variety of sources: (1) from arginine via NOS catalysis, (2) Substitution from ornithine by decomposition of proline or glutamine/glutamic acid, (3) from ADMA, it is catalyzed by dimethylarginine dimethylamine hydrolase (DDAH)^83^. Glutamine in tiny intestinal epithelial cells produce citrulline, which is subsequently converted to arginine with the assistance of renal tubular cells. A randomized controlled clinical trial manifested that glutamine supplementation during the perioperative period attenuated the impaired renal arginine synthesis after open aortic surgery and compensated for the suppressive effects of ischemia-reperfusion injury^79^. However, to date there is no definitive evidence to assess whether glutamine supplementation affects AAD, and there are reports that excessive glutamine circulation to the TCA cycle leads to an abnormal pro-inflammatory response^84^. It is questionable whether glutamine induces an inflammatory response to the vessel wall in AAD. Moreover, protein arginine methyltransferase 1 (Prmt1) is the predominant enzyme that catalyzes the asymmetric arginine dimethylation of proteins, which is the source of ADMA. Prmt1 induces the switch from contractile to synthetic VSMC by regulating myocardin levels and asymmetric dimethyl-H4R3 histone modifications, leading to downregulation of VSMC contractile gene expression and upregulation of synthetic gene expression, resulting in the formation of AAD^85^.

The recoupling of eNOS alleviated expansion of aortic roots and abdominal aortas, then animal models of endothelial dysfunction and eNOS uncoupling increased the rate of AAA formation and increased eNOS augmented the risk of aortic dissection. The increase of eNOS-derived ROS and the decrease of NO production may be the main causes of endothelial dysfunction in AAD^86^, ^87^. Nw-nitro-L-arginine methylester (L-NAME), serving as NOS inhibitor, induced NO suppression to cause severe endothelial dysfunction, which could accelerate the onset of dissection but not aneurysm^88^. At the same time, a randomized, single-blind, multi-centre, and placebo-controlled trial indicated that patients receiving perindopril which contained 10 mg arginine showed similar blood pressure lowering effects as amlodipine, but perindopril did not affect the overall rate of AAA growth during 2 years of follow-up^89^. Improving NO bioavailability might suggest a potential treatment strategy in AAD, and arginine metabolic pathway may be the link between NO bioavailability and AAD (Figure 3B).

### Tryptophan

Tryptophan (Trp) is another essential amino acid that can be obtained from dietary consumption, which has been proven to influence AAD. Trp is essential for protein synthesis and therefore for normal cellular homeostasis. There are two pathways that process Trp into other metabolites, including the 5-hydroxytryptamine (5-HT) pathway and the kynurenine (Kyn) pathway. Hepatic tryptophan-2,3-dioxygenase (TDO) maintains physiological concentrations of Trp and Kyn at modulated levels via the kynurenine pathway (KP)^90^. It has been found that tryptophan catabolic processes to acetyl-CoA, tryptophan catabolic processes to kynurenine, and tryptophan catabolic process were enriched in the human AAA group^91^.

During tryptophan catabolism, tryptophan is further metabolized to L-kynurenine (Kyn) via indoleamine 2,3-dioxygenases 1 and 2 (IDO1 and IDO2) and TDO to produce N-formylkynurenine. Kyn is further catabolized to the active metabolites 3-hydroxykynurenine (3HK), 3-hydroxyaminobromic acid (3-HAA), kynurenine acid (KA), quinolinic acid (QA), and the essential pyridine nucleotide end product nicotinamide adenine dinucleotide+ (NAD+)^92^. The accumulating evidence demonstrates that Trp has an essential contribution to the execution of AAD development. It has been identified that kynurenine pathway activity is enhanced in aortic atherosclerotic aneurysm. Metabolite levels of tryptophan, kynurenine, and QA were enhanced in the walls of the aneurysm, and inhibition of kynurenine increased IDO1 levels in cultured macrophage^93^. Furthermore, the expression of 3-HAA, IDO, and kynureninase were obviously augmented in human AAA samples, IDO deletion in vivo restrained AngII-induced AAA in Apoe^-/-^ mice and low density lipoprotein receptor-deficient mice fed with high fat diet^94^, ^95^. Mechanistically, 3-HAA contributes to increased NF-κB-Mediated MMP2 expression in HASMCs, leading to promote phenotypic switching of VSMCS which accelerated angiotensin II-induced AAA formation in mice^94^. On the other hand, ECs dysfunction and apoptosis are important pathophysiological processes in the development of AAD, and 3-HK has been shown to induce endothelial cell apoptosis and endothelial dysfunction by increasing NAD(P)H oxidase (NOX)-derived ROS. Therefore, inhibition of 3-HK formation may be a prospective therapeutic intervention to prevent AAD^96^ (Figure 3C). Overall, a causal relationship between Kyn pathway activation and AAD development has been established, suggesting that Trp-derived metabolites may be diagnostic biomarkers for AAD and may have the potential to be therapeutic for AAD.

### Taurine

Taurine, mainly derived from seafood such as shellfish, is a non-protein amino acid with a distinctive sulfonic acid composition^97^. Evidence suggests that taurine affects a variety of cellular functions, including osmolarity, oxidation, and bile acid binding. At the same time, taurine has anti-inflammatory effects and has been proved to have the benefit of suppressing the renin-angiotensin system in CVDs. Populations with higher meat intake exhibited higher CVD related mortality compared to those with higher seafood intake^98^. It is possible that the amount of taurine in seafood is much higher than that in meat, suggesting that taurine fulfill an essential function in preventing CVD.

Research has identified a correlation between taurine and vascular dysfunction. Vascular dysfunction was attenuated in type 2 diabetes patients after taurine supplementation in a clinical study, indicating that taurine therapy reverses endothelial dysfunction, which is the central pathway for aortic aneurysms and aortic dissection. Supplementation with taurine is proved to alleviate myeloperoxidase-mediated oxidative stress, leading to the mitigation of AAA formation in mice^99^. In the aortic valve interstitial cells (AVICs) isolated from patients with type A dissection without leaflet disease, taurine attenuated PCM-induced osteogenic differentiation of AVICs by reducing alkaline phosphatase (ALP) activity in a dose-dependent manner^100^. Therefore, taurine supplementation may elicit a beneficial role in the progression of AAD and further investigations are required to confirm whether taurine supplementation is a feasible adjunct treatment of AAD.

## Lipid metabolism in AADs

### Polyunsaturated fatty acids (PUFA)

Fatty acids are the major components of dietary fat, which is composed mainly of saturated fatty acids (SFA) and polyunsaturated fatty acids (PUFA)^101^. An initial observational study demonstrated lower mortality from CVDs in people with a high intake of ω -3 PUFA102,103 and thus attracted attention to the cardiovascular protective effects of PUFA.^102^, ^103^.

**Figure 4:**
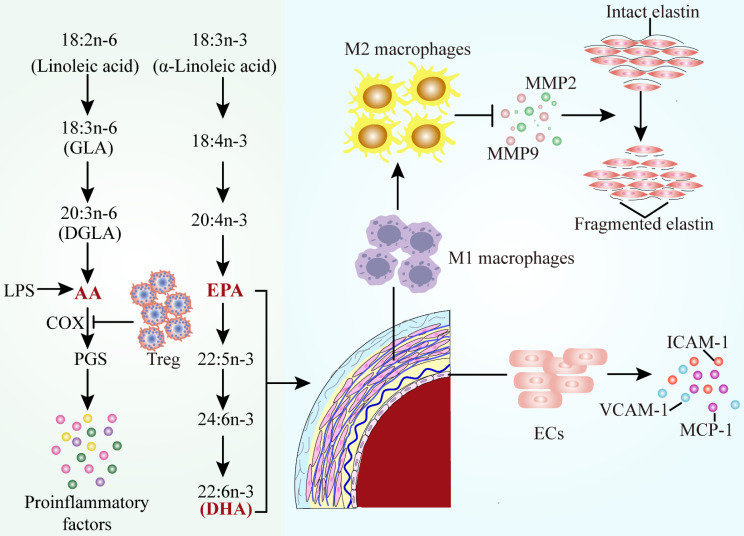
** Alterations of polyunsaturated fatty acids metabolic pathways in AAD.** The classification, formation of polyunsaturated fatty acids and their function in AAD. Decreased concentrations of AA, EPA and DHA lead to the occurrence of proinflammatory events in AAD. Abbreviations: AA, arachidonic acid; DHA, docosahexaenoic acid; EPA, eicosapentaenoic acid; ECs, endothelial cells; Treg, regulatory T cells; GLA, gamma linolenic acid; DGLA, dihomo gamma linolenic acid; PGs, prostaglandins; COX, cyclooxygenase; LPS, lipopolysaccharide; MMP2, matrix metalloproteinase 2; MMP9, matrix metalloproteinase 9; VCAM-1, vascular cell adhesion molecule 1; ICAM-1, intercellular adhesion factor 1; MCP-1, monocyte chemoattractant protein 1.

PUFAs are further divided into the omega-6 PUFAs and the omega-3 PUFAs according to the location and function of the double bond. Linoleic acid and arachidonic acid (AA) belong to the ω-6 PUFAs, while linolenic acid, docosahexaenoic acid (DHA) and eicosapentaenoic acid (EPA) belong to the ω-3 PUFAs^104^ (Figure 4). The Mendelian randomization analysis of FA genetic data illustrated the role of circulating PUFAs on cardiovascular diseases risk^105^. Higher plasma alpha-linolenic acid, linoleic acid, and oleic acid levels were correlated with a lower probability of large artery stroke and venous thromboembolism, while higher levels of arachidonic acid and stearic acid were associated with a higher probability of occurrence^106^. Higher genetically predicted DHA levels were associated with higher risk of CVDs endpoints, and the levels of DHA were risk factors for AAD.

AAD are chronic inflammatory diseases, which are initiated by the accumulated macrophages, release of inflammatory factors, and oxidative stress. In consequence, pro-inflammatory AA seems to play an important role in AAD. The pro-inflammatory response observed in AAD could be due to the lack of anti-inflammatory AA results^107^. Therefore, it is necessary to investigate whether the combined application of PUFA is more suitable for the prevention of AAD in humans.

#### Arachidonic Acid Metabolism

AA is one of the most abundant PUFAs in the human body and serves as a component of cell membrane phospholipid bilayers, an inflammatory mediator and a vasodilator or constrictor. Endogenous AA is mainly involved in the production of various bioactive derivatives of arachidonic acid, which can be metabolized by three enzyme systems, COX, cytochrome P450 (CYP) enzymes and lipoxygenase (LOX)^108^. The imbalance between thromboxane A2 (TXA2) and prostacyclin synthesis is attributed to differential expression of isoform proteins of COXs, which suggests the alteration in AA metabolism. It has been observed that the concentration of arachidonic acid is decreased, which is connected with an enhancement of COX2, CYP450 and 5-LOX, leading to the formation of the aortic aneurysm^109^. The RBC proportion of arachidonic acid increased prevalence of AAA and the risk of surgical repair, indicating the potential of AA served as a prognostic biomarker. It has been also reported that the level of AA and thrombin mediated platelet reactivity could represent a switch of vascular inflammation in patients after major vascular surgery^110^.

COX-2 can catalyse converting of AA to prostaglandins (PGs), modulating the expression of pro-inflammatory chemokines to regulate cell functions. COXs and PGs are considerable inflammatory mediators which are associated with AAD^111^. COX-2 is generally not detectable in most physiological states, whereas it can be rapidly triggered in response to various inflammatory stimuli (e.g. LPS or Ang II) and COX-2 expression is strongly characterized in AAD^112^. Compared to healthy individuals, COX-2 expression is visibly increased in AAD patients^113^. COX-2 knockdown significantly alleviated the incidence and severity of Ang II-induced AAA in mice. Moreover, Liu et al. detected that CD4+CD25+ regulatory T cells (Tregs) adoptive transferred to suppress the level of COX-2 in AAA aortic tissues, indicating that Tregs protected against AAA by suppression of the COX-2 expression^114^.

#### Omega -3 Polyunsaturated Fatty Acid

Interest in omega-3 (ω-3) PUFAs has escalated in recent years because of their various roles in health promotion and disease risk reduction. ω-3 PUFAs include EPA, DHA, α-linolenic acid (ALA), stearidonic acid (SDA), and docosapentaenoic acid (DPA). ω-3 PUFAs with health benefits ascribed mainly to EPA and DHA, which are primarily obtained from the diet such as the body lipids of fatty fish and the liver of white lean fish^115^. Multiple studies have been conducted on the effects of ω-3 PUFAs on dominating cardiovascular diseases, such as MI, stroke, AF, coronary heart disease, heart failure (HF), and AAD^116^.

A randomized controlled trial demonstrated that erythrocytes from patients with AAA exhibit an alternating FA profile characterized by an anomalous ratio of n-6 FAs with inflammatory state, reflecting the effects of systemic inflammation and oxidative stress. However, the inflammatory parameters and n-6 fatty acid status were significantly improved afterω-3 PUFA supplementation, indicating that dietary fatty acids conduct a potential therapeutic intervention for AAA^117^. Additionally, supplementation of ApoE^-/-^ mice with a diet high in n-3 PUFA content protected the mice against pro-inflammatory and oxidative stress responses following short-term infusion with Ang II^118^. ω-3 PUFA also commonly occurr in tree nuts. Previous studies have reported that administrating ω-3 PUFAs can limit AAA development and severity within experimental animal models^119^. Based on this previous experimental evidence, it was investigated that a diet enriched with tree nuts could limit the development and severity of AAA^120^.

EPA plays a protective role in abdominal aortic aneurysm, animal models and macrophages by multiple pathways. In the patients with surgical repair of AAA, EPA/AA ratio was lower than those in healthy Japanese subject, which was negatively correlated with the maximum AAA diameter. Low serum EPA levels and EPA/AA ratio were associated with the size and growth rate of AAA^121^. The protective effects and underlying mechanisms of EPA in AAA have been identified (Figure 4): (1) increased anti-inflammatory property of macrophages due to lessened inflammatory factor such as MMP2 and MMP9 levels^122^-^124^ (2) attenuated of mesenchymal stem cell (MSC) dysfunctions^125^ (3) suppressed the weakening of the vascular wall by inhibiting the elastin fiber degradation caused by nicotine^126^ (4) inhibition of endothelial cell-mediated inflammatory factors^107^. AAA is associated with inflammation and oxidative stress, the latter of which contributes to activation of macrophages, a prominent cell type in AAA. Meanwhile, a recent study found that DHA decreased the concentration of TNF-α and interleukin-6 (IL-6) in macrophage supernatants and increased glutathione peroxidase activity and heme oxygenase-1 expression. The improvements in macrophage oxidative stress status serve as a stimulus for further investigation of DHA in patients with AAA^127^.

#### Nitro-fatty acids (NO2-FAs)

Nitro-fatty acids (NO2-FAs), electrophilic lipid molecules, are endogenously synthesized via the reaction of unsaturated fatty acids (USFAs) with NO or nitrite-derived nitrogen dioxide free radicals^128^. NO2-OA has been shown to confer substantial cardioprotection through supressing the activation of NF-κB. Given that NO2-FAs are preferred in terms of synthetic properties and stability, it emerged in two Phase II studies including patients with PAH (NCT03449524) and focal segmental glomerulosclerosis (NCT03422510). Importantly, NO2-OA has been investigated to attenuate ascending aortic dilation and wall stiffening in Marfan syndrome (MFS) mice by inhibiting the overactivation of ERK1/2, Smad2 and NF-κB in the aorta, thus reducing elastin fragmentation, cell apoptosis and collagen deposition induced by matrix metalloproteinase 2^129^. Correspondingly, NO2-OA exerts a protective effect on the observable decrease of extracellular matrix degradation, inflammatory cytokine release, and macrophage infiltration in AAA model^129^. With the current studies, the data might expedite the use of NO2-OA for AAA therapy.

### Other Lipid Metabolites in AADs

It is well established that oxidative stress and inflammation are the two main mechanisms leading to AAD, and the modification of cell membrane phospholipids has been proved in AAD^130^. Lysophosphatidylcholines (LPCs) and sphingolipids were significantly altered in AAD patients compared with healthy controls. Furthermore, three sphingolipids, including sphingosine, phytosphingosine, and ceramide, were specifically significantly decreased in Stanford type A AAD patients. It can be utilized as potential biomarkers for the diagnosis of AAD and to distinguish Stanford type A from Stanford type B^131^. Under oxidative stress, PUFA-containing phospholipids and cholesterol esters in cellular membrane and lipoproteins can be readily oxidized through a free radical-induced lipid peroxidation (LPO) process to form a complex mixture of oxidation products. The association of serum lipid peroxidation products with disease severity in AAA patients has been determined^132^. Macrophages in the vascular wall could amplificate the local inflammatory response by secreting proinflammatory cytokines and degradation of ECM by producing proteases to promote the occurrence of AAA. GW4869, an inhibitor of sphingomyelinase, ameliorated extracellular vesicles (EVs) generated by T lymphocytes and reduced macrophage migration and infiltration, thereby inhibiting the development of AAA in elastase-deficient mice^133^. The presence of lipid-protein interactions in the membrane provides substrates lysophosphatidylcholine (LPC) and lysophosphatidic acid (LPA). Autotaxin (ATX) directly interacting with integrin on the target cell surface to regulate cell function as a secreted protein, lysophosphatidase D (lysoPLD) which catalyzes the conversion of LPA of LPC based on its catalytic phosphodiesterase (PDE) domain^134^. Plasma ATX concentration is up-regulated in elastase-induced AAA mouse model, ATX interacting with T cells by binding to integrin α4 activated the FAK/Src-RhoA signaling pathway to promote T cell migration, which in turn recruits T cells into the vessel wall and induces vascular inflammation, thereby accelerating the pathogenesis of AAA^135^. Additionally, bioactive lipids play a central role in AAA by regulating coagulation and its associated inflammation. Coagulation factors assemble on phospholipid (PL) membranes to induce thrombin generation to promote hemostasis occurrence, aminoPL (aPL) and enzymatically oxidized PL (eoxPL) work together to achieve coagulation^136^. Mice with AAA were shown to have increased intravascular coagulation and formation of large amounts of eoxPL. Instead of activating circulating clotting factor consumption and depletion to reduce AAA, coagulation is kept away from the vessel wall due to eoxPL deficiency^137^. Complex interactions between bleeding and thrombosis through the vessel wall or in the circulation can drive or prevent AAA development, providing new insights into AAA pathogenesis and treatment. Importantly, plasma phospholipid transfer protein (PLTP), another cardiovascular risk factor, has been shown to regulate the coagulation process. Mice with PLTP deficiency showed reduced VSMC depletion, elastin degradation and macrophage infiltration, and reduced expression and release of tissue matrix metalloproteinase. PLTP deficiency was associated with a significantly lower incidence and less severe degree of AAA expansion. PLTP may be of particular clinical relevance due to its dual proinflammatory and hyperlipidemic effects. However, it remains to be determined whether the PLTP blockade may constitute a relevant approach for AAA prevention^138^. Indeed, HDL is known to display beneficial antielastase activity^139^, and plasma HDL cholesterol levels are reported to be inversely associated with the incidence of AAA^140^.

## TCA Cycle and Electron Transport Chain (ETC)

Cellular metabolism mainly involves the utilization of carbohydrates, proteins, and fats for energy synthesis, and when metabolic processes are used to produce energy, the final step is oxidative phosphorylation^141^. Molecules from these processes are used in the tricarboxylic acid (TCA) cycle to generate substrates that enter the ETC for oxidative phosphorylation. The ETC, located in the inner mitochondrial membrane, receives electrons donated by NADH and FADH2 produced by the TCA cycle at complex I (NADH: ubiquinone oxidoreductase) or complex II (succinate dehydrogenase), respectively^142^, generating cellular ATP. In brief, Complexes I and II, with additional the flavoprotein-ubiquinone oxidoreductase, transfer electrons to ubiquinone (coenzyme Q). Subsequently, electrons are transferred sequentially to Complex III (coenzyme Q: cytochrome c reductase), cytochrome c, Complex IV, and eventually to molecular oxygen, the terminal electron acceptor. Complex V consists of two distinct domains, including an extracellular domain (known as F1) and a transmembrane domain (known as FO). Proton movement from the membrane gap through FO is coupled to rotation, resulting in the synthesis of ATP by the addition of a phosphate and ADP at the F1 site, which ultimately leads to produce ATP^143^.

There has been evidence that ETC is defective in AAD. A microarray study identified 47 significantly differentially expressed genes in AAA patients compared with controls. GO functional analysis revealed that these genes play important roles in electron transport chain^144^. Moreover, the protein level of Complexes I through IV was significantly increased in Fibulin4^R/R^ aortas compared with Fibulin-4^+/+^, whereas Complex V had no significant change. In Fibulin-4^R/R^ mice, the extracellular matrix protein Fibulin-4 was significantly reduced, which results in decreased blood ketone and hepatic fatty acid levels, indicating dysregulated metabolism to promote thoracic aortic aneurysm formation^145^. It has been observed that CR exacerbated Ang II-induced AAA formation in p53^-/-^ mice. Notably, knockout of p53 promoted Ang II-induced AAA via inhibiting the expression of cytochrome C oxidase assembly protein 2 (SCO2) which is a component of the complex IV.^40^ Accordingly, the enzymatic activities of citrate synthase and cytochrome C oxidase, serving as biomarkers of mitochondrial respiratory chain functionality, were significantly reduced in the AAA aorta^146^. Consistently, the transcriptomics and metabolic analysis of aortas from an MFS mouse model revealed that mitochondrial complexes were reduced. Supplementation with mitochondrial metabolism NAD precursor nicotinamide riboside improves mitochondrial function to reverse aortic aneurysm^147^. Taken together, the absence of ETC might aggravate the occurrence of AAA, suggesting mitochondrial boosting strategies as a potential treatment to managing aortic aneurysms and therapies targeting ETC could benefit patients with AAA.

**Figure 5:**
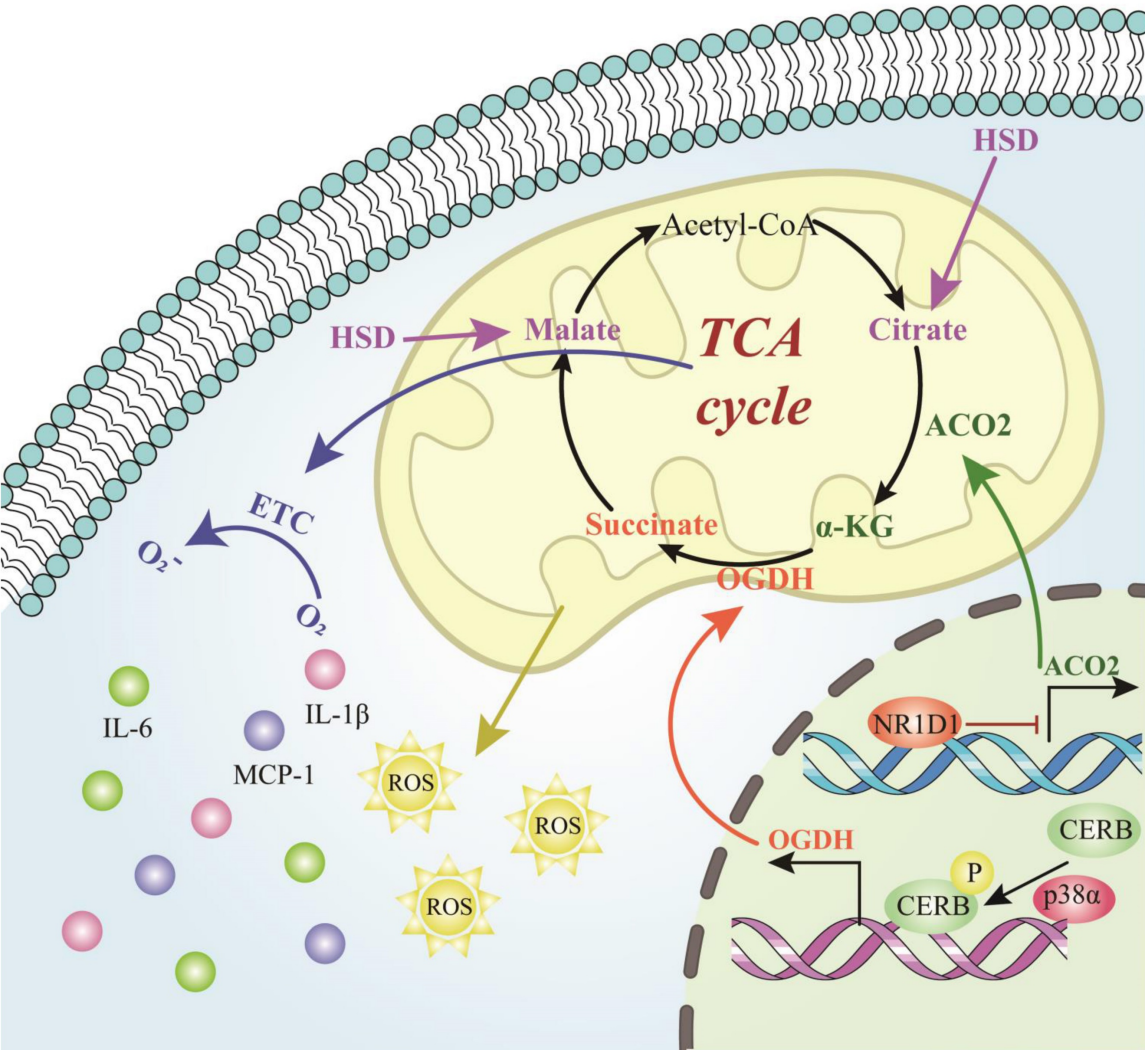
** Role of tricarboxylic acid cycle-related cellular metabolites in AAD.** The process of the intracellular tricarboxylic acid cycle and the role of the corresponding metabolites in AAD. The process figures those molecules and reactions found in the TCA cycle in mitochondria. Abbreviations: TCA cycle, tricarboxylic acid cycle; HSD, high salt diet; α-KG, alpha‑ketoglutarate; ACO2, aconitase-2; Acetyl-CoA, acetyl coenzyme A; OGDH, oxoglutarate dehydrogenase; ROS, reactive oxygen species; ETC, electron transport chain; IL-6, interleukin-6; IL-1β, interleukin-1β; MCP-1, monocyte chemoattractant protein 1; NR1D1, NR subfamily 1 group D member 1; CREB, cyclic adenosine monophosphate-responsive element-binding protein 1.

The TCA cycle, also known as the citric acid cycle or the Krebs cycle, is a series of reactions in a closed loop that forms a metabolic engine within cells (Figure 5). While the TCA cycle is vital of the oxidation of substrates for energy production (ATP), TCA cycle metabolites have been demonstrated to serve as signaling molecules with functions regulating chromatin modifications, DNA methylation, the hypoxic response, and immunity, leading to change cell function and fate in diverse diseases^148^. Sodium pyruvate (SP), a substrate of the TCA cycle, has been demonstrated to be the potential target for hypoxic-ischemic encephalopathy. SP improved cerebral metabolism via maintaining ATP levels and inhibiting intracellular reactive oxygen species (ROS) levels to alleviate brain damage and enhance neurological performance^149^. Mechanistically, it has been found that visnagin bound to mitochondrial malate dehydrogenase (MDH2) which was a key enzyme in the TCA cycle, resulting in reducing doxorubicin-induced cardiomyocytes apoptosis and increasing cardiac contractility in mice^150^. Furthermore, a maladaptive increase in anaplerosis via malic enzyme 1 (ME1) in TCA is associated with reduced GSH content. In hypertrophic rat hearts, inhibition of ME1 expression can reduce pyruvate carboxylation, reduce lactate accumulation, and restore GSH content, thereby normalizing hypertrophy^151^.

Hypertension is an important risk factor for aortic aneurysm and aortic dissection. When the body is under the level of hypertension for a long time, it will lead to the change of blood flow of the patient, which in turn can cause the change of the ratio of elastic fibers to collagen and morphology of the aortic wall, increase the shear stress of the blood vessel, increase the stiffness of the blood vessel wall, and eventually cause AAD^152^. High salt diet (HSD) significantly weakened the TCA cycle, and attenuated the antioxidant system in the renal medulla, which finally contributed to salt-sensitive hypertension^153^. Metabolomic profiling was performed that three serous TCA-cycle-associated compounds, including decreased malic acid and citric acid, as well increased fumaric acid were differentially detected in salt-sensitive rats treated with HSD^153^. Alpha‑ketoglutarate (α-KG) has been reported to exert significant impacts on AAA. α-KG significantly suppressed the aneurysmal dilation by attenuating the oxidative stress, macrophage infiltration, elastin degradation and collagen fibers remodeling via inhibiting PXDN/HOCL/ERK signaling pathways^154^. Moreover, NR1D1 contributes to AAA formation by targeting the mitochondrial TCA cycle enzyme aconitase-2 (ACO2). The activity of the key TCA cycle enzyme ACO2 in both human and mouse AAA samples were decreased, which mediated the negative effects of NR1D1 on mitochondrial function and AAA formation. Supplementation with α-KG, a downstream metabolite of ACO2, rescued mitochondrial dysfunction and protected against AAA formation^155^. Additionally, untargeted metabolomics identifies succinate as a biomarker and therapeutic target in AAD. Plasma succinate concentrations were increased in patients with AAD which can be used as a biomarker for diagnosis and allow reliable differentiation from AMI and PE when patients present with chest pain. Mechanistically, oxoglutarate dehydrogenase (OGDH) is a key upstream TCA enzyme for direct synthesis of succinate, inhibition of OGDH reduces the expression of inflammatory factors in macrophages to increase the mitochondrial ROS level in the vasculature, further aggravated AAD formation targeting p38a-CREB-OGDH axis^9^. Collectively, the results of the present study provided evidence that the TCA cycle metabolites played powerful parts in AAD formation and progression and were identified as potential novel targets for AAD treatment.

## The Role of Other AAD-associated Metabolites

It is well established that in addition to the AAD-associated metabolites we have described above, there are numerous additional metabolites that play a crucial role in the pathogenesis of AAD.

Sex is a prominent risk factor for AAA, and Ang II induces AAA formation to a greater degree in male than in female mice. It has been found that a cytochrome P450 1B1generated metabolite of testosterone, 6βhydroxytestosterone (6βOHT), contributed to Ang II-induced AAA development in male mice, which provides a bridge between sex and metabolites in AAD pathogenesis^156^. Additionally, vitamin D deficiency could exacerbate AA development via regulating the genes involved in extracellular matrix remodeling^157^.

Uric acid (UA) is a purine derivative and a well-established CVD risk marker. Serum uric acid is associated with AD in Chinese men^158^, ^159^, and it can serve as an indicator for the differential diagnosis of AD and CAD or a marker of oxidative stress in dilatation of the ascending aorta^160^, ^161^. It has been speculated that a non-linear correlation was determined between serum UA and in-hospital mortality of patients with acute type A aortic dissection^162^. When serum uric acid > 260 µmol/L, it showed a positive correlation with in-hospital mortality. Further clinical studies are necessary to investigate whether UA is an independent risk factor for AAD.

Polyphenols are naturally occurring compounds found largely in fruits, vegetables, cereals, and beverages. Numerous polyphenols including resveratrol and curcumin have been shown to be associated with AAD. Resveratrol attenuated AD by increasing endothelial barrier function through the SIRT1 pathway^163^. Meanwhile, curcumin, radix astragali, resveratrol, Baicalein, and Salvianolic acid have been demonstrated to be potential treatments for AAA^164^.

## Metabolic profile and potential therapies for AAD

Previous studies have shown that the metabolic profile of AAD patients is significantly different from that of healthy participants or patients with other diseases. Therefore, we summarized the metabolic profile of AAD patients to contribute to provide potential targets for AAD clinical treatment (Table 2). Overall, the main observations were that the AAs and phosphatidylcholine levels were decreased^165^, and the metabolism of sphingolipids, lysophospha-tidylcholine, cholesterol, and acylcarnitine was disturbed in AAA patients compared with healthy subjects^166^. A combination of salicylic acid, pregnenolone sulfate, 5-Lglutamyl-L-alanine, and 3-hexanone with mevalonic acid could serve as a biomarker to distinguish AAA and atherosclerosis^167^. On the other hand, the alterations of most lipid metabolism (phosphatidylcholine, lysophosphatidylcholines, triacylglycerols, fatty acids, acylcarnitines etc.)^10^, ^131^, ^168^ polyunsaturated fatty acids metabolism (EPA and DHA) and ceramide metabolism^23^ were further observed in AD patients but not in healthy individuals. Meanwhile, AD patients also show characteristic metabolic profiles compared to other diseases. It is well established that hypertension and CHD are important risk factors for AD, therefore, the level N1-acetyl-N2-formyl-5-methoxykynuramine (AFMK), ergothioneine, glycerophosphocholine, and Sphingofungin B in the peripheral blood of the AD patients were distinctly different compared to hypertension patients^169^. Plasma aminograms were significantly altered in patients with AD compared with CHD, especially in acute AD. Interestingly, among AD patients, different types of AD also present differential metabolic profiles. The contents of glutamate and phenylalanine were significantly changed in acute AD patients comparing with chronic AD patients^170^. Sphingolipids, including sphinganine, phytosphingosine, and ceramide, were reduced in the Stanford type A AAD patients, but not in the Stanford type B AAD patients^131^. As for the differences in metabolic profiles between AA and AD, Zhang et al. found 138 differential metabolites which were mainly involved in the galactose pathway, while sphingolipids, especially its core metabolite C18 ceramide, were significantly differentiated in thoracic aortic dissection (TAD) patients but not in thoracic aortic aneurysm (TAA) patients, which was an important step for better understanding the differences between AA and AD^171^. The above studies suggest that amino acid and lipid metabolism were expected to exploit a novel and non-invasive diagnosis for AAD.

In addition to the role of metabolites as biomarkers in the diagnosis of AAD, the metabolic changes of AAD suggest a novel metabolic pathway for the treatment of AAD. Targeting metabolism has been proposed in the treatment of AAD and shows some achievement. Chronic inflammation of the aorta has been reported to underlie AAA pathology, with infiltrating leukocytes producing cytokines and proteases that ultimately lead to deterioration of the vessel wall^172^. To date, anti-inflammatory drugs and immunosuppressive drugs have been demonstrated to exert on the inhibition of aneurysm formation. For example, celecoxib, sirolimus, and cyclosporine A have been shown to prevent aneurysm formation potently in the animal aneurysm models, whereas hydrocortisone induced aortic rupture and aneurysm formation in mice^173^-^175^. Notably, Azathioprine (Aza), an immunosuppressive drug, could promote metabolite 6-MP to modulate the activation of c-Jun-terminal-N-kinase (JNK) in ECs, leading to suppress aneurysm progression^176^. Targetting amino acid metabolism and blocking Hcy accumulation can also be viable treatments for AAD. OT-58, the most representative drug for HHcy treatment, has entered phase 1/2 clinical trials and might play a crucial role in the future treatment of AAA^177^-^179^. At the same time, a low methionine diet combined with vitamins B6, B12, and FA can reduce serum Hcy concentration in HHcy patients with AAA, which may have a protective effect on AAA^180^, ^181^. Several IDO inhibitors which fulfill a function in Tryptophan, such as Indoximod, Epacadostat, and Navoximod, have been approved for patients in anti-tumor therapy^182^, but there is a lack of research to assess the application of these drugs on AAA. Studies have found that Glycine (Gly) has protective effects on lowering blood pressure^183^, ^184^, and supplementing the diet with an appropriate dose of Gly could prevent cardiovascular diseases, suggesting that Gly may play a powerful part in the treatment of AAD by controlling blood pressure which is an important risk factor for AAD.

Based on previous studies on the relationship between lipid metabolism and AAA, it is possible to develop drugs for the prevention and treatment of AAA from the perspective of regulating lipid metabolism. High density lipoprotein (HDL) enhanced the level of annexin A1 (Anxa1) to inhibit endothelial cell inflammation, while Anxa1 has been shown to restrain the occurrence of acute aortic dissection by inhibiting the phenotypic transformation of vascular smooth muscle cells, suggesting that there may be drug targets for the treatment of AAD that target the inflammation response to HDL^185^, ^186^. Furthermore, previous clinical studies reported that proprotein convertase subtilisin-kexin type 9 (PCSK9) inhibitors can reduce AAA risk, and PCSK9 inhibitors can reduce low-density lipoprotein cholesterol (LDL) by inhibiting degradation of the LDL receptor, whereas mutations in PCSK9 aggravated AAA development in mice^187^. Although their clinical effect on AAA has not been tested, it provides a new perspective for the clinical treatment of AAD.

Therefore, the risk of AAD formation can be assessed clinically by detecting the levels of corresponding metabolites in plasma or serum. For patients with a high risk of AAD, such as the elderly and hypertension, dietary advice can also be provided to achieve the purpose of early prevention. In addition, although the research on the recognized therapeutic drugs for AAD is still a wastland to be reclaimed, the metabolism-related experimental animal studies have provided numerous new clues for potential drug targets.

## Conclusion and perspectives

Increasing evidence indicates a link between the metabolomics and AAD incidence. Multiple metabolites interact with the host through a variety of pathways and play an indispensable role in the occurrence and development of diseases. Altered metabolite composition or different metabolite concentrations might be responsible for AAD risk and its associated pathological changes. Thus, novel therapeutic targets for preventing and treating AAD have been developed harnessing the potential use of metabolites.

Metabolic abnormalities are found in human AAD and animal models of AAD. The metabolism of gut microbiota-derived secondary metabolites is characterized by increased circulating levels of TMAO and LPS, decreased levels of bile acids including UDCA and TUDCA, and decreased short-chain fatty acids such as butyrate. The above characteristics indicate that the differential expression of secondary metabolites caused by intestinal flora disorder are engaged in the pathological process of AAD, which makes it possible to target AAD therapy against intestinal flora. In addition, the differentially expressed primary metabolites were observed to be mainly involved in amino acids, phosphatidylcholines, galactose, ceramide, and polyunsaturated FAs metabolic pathways in AAD,^131^, ^165^, ^171^ which suggested potential disorders of lipolysis, FA synthesis, and amino acids transport in patients suffering from AAD. Arginine metabolism, which links NO production and TCA cycle, has a key role linking the vasoconstrictive phenotype of the disease with the metabolic abnormalities.^188^ Moreover, changes in TCA cycle intermediates can contribute to regulate ROS generation, driving alterations in the functions of key genes of AAD. Decreased levels of AA and ceramide metabolism disturbance in the AAA patients supply further evidence of the inflammatory pathway in the pathogenesis of AAD, such as NLRP3 pathway.^189^, ^190^ Therefore, therapeutic approaches targeting metabolic disorders might be relevant to address many cellular pathologic phenotypes in AAD.

The existing research suggests that efforts are being made to advance the potential application of metabolites in CVD. However, limitations exist in studying metabolites in AAD. First of all, gut microbiota plays a crucial role in regulating gut microbiota-derived secondary metabolites. Identifying specific bacteria present in AAD can help clarify the contribution of gut microbiota-derived metabolites to disease progression. Nevertheless, research on the gut microbiota profile of AAD remains limited. Secondly, while most current studies focus on exploring the metabolic profile of AAD, including differential metabolites and changes in metabolite concentrations, they lack elucidation of the mechanism by which metabolites cause AAD. Future studies should therefore prioritize investigating signaling pathways mediated by metabolites and their downstream functional consequences. Thirdly, there is an urgent need for personalized metabolic profiling. The analysis of spatial metabolic biomarkers in individual AAD patients, as opposed to other cardiovascular diseases, may significantly contribute to this endeavor. Fourthly, the clinical application of metabolites in the treatment of AAD is hindered by a lack of safe and effective conversion pathways. It seems that inhibitors targeting metabolite production and dietary supplementation are effective ways to implement, but large-scale population validation is lacking. Finally, most studies focus on the effects of various metabolites on AA, and there are few studies in this field of AD, and we speculated that it may be caused by the characteristics of acute onset and elevated mortality of AD. However, it is of great significance to strengthen the study of metabolomics of AD to improve the metabolic profile of aortic diseases.

The focus of future studies is to figure out how metabolites lead to AAD at the mechanistic level. This involves characterizing metabolites, microbes, and microbial-host interactions within complex microbiota ecosystems. Recognizing the role of gut microbiota and metabolites in both health maintenance and disease susceptibility offers a novel perspective that broadens our approach to AAD research and therapeutic interventions. Additionally, it is important to investigate potential genetic factors that may influence susceptibility to disease in combination with specific nutrient depletion/excess and the functional capacity of the microbiota. Finally, prospective cohort studies can bring metabolite research and dietary interventions into clinical practice. By measuring metabolic profiles in blood or urine to guide appropriate dietary recommendations and deliver targeted interventions, more translational findings will be achieved. These interventions may take various forms in the future, including dietary advice, inhibitors or agonists of metabolites, nonlethal microbial inhibitors or probiotics. Another alternative approach is to modulate gut microbiota to achieve changes in gut microbiota-derived metabolites, which is an active area of research.

Based on the above limitations and perspectives, it is beneficial to address the question of how metabolic abnormalities develop in AAD in the future, thus determining which pathways are ideal therapeutic targets. Fully understanding metabolic abnormalities of AAD over the course of the disease is the essential next step for developing therapies.

## Figures and Tables

**Table 1:** The association between Hcy and patients with AAD.

Reference	Cohort size	Type of AAD	Cohort background	Altering catabolites in AAD
Eftihia et al^191^	31 aortic dissection (AD) patients, 30 aortic aneurysm (AA) patients, and 20 controls	AAD	Greece	Hcy↑ Folate ↓
Sakshi et al^192^	142 abdominal aortic aneurysm (AAA) patients and 279 controls	AAA	Sweden	Hcy↑
Yuen et al^193^	12,203 men including 318 AAA patients	AAA	Australia	tHcy↑
Deng et al^194^ Liu et al^195^	73 AAA patients and 219 controls, 155 AAA patients and 310 controls	AAA	China	HHcy is an independent risk factor for AAA
Crystal et al^196^	30 AAA patients and 31 controls	AAA	China	Hcy in AAA tissues↑
Aldona et al^197^	36 patients treated for AAA repair	AAA	Poland	Hcy in damaged arterial wall↑

**Table 2:** Current metabolic profilings of AAD.

Reference	Origin	Description	Study size	Substrate	Type of AAD	Metabolic profile and potential metabolic treatment targets
Lieberg et al^165^	Estonia	Comparison of plasma metabolites between patients with AAA and aorta-healthy controls	AAA with fast yearly growth rate:39 AAA with slow yearly growth rate:39 Healthy subjects:79	Plasma	AAA	Amino acids and phosphatidylcholines metabolic pathway
Ji et al^167^	China	Comparison of plasma metabolites between patients with AAA and AS patients	AAA patients:32 AS patients:32	Plasma	AAA	2-Deoxy-D-ribose (2dDR)
Zeng et al^10^	China	Comparison of serum metabolites between patients with Stanford Type A AAD and healthy individuals	Type A aortic dissection (TAAD) patients:19 Healthy individuals:20	Serum	AD	Phosphatidylcholine metabolic pathway
Zhou et al^131^	China	Comparison of plasma metabolites between acute aortic dissection and healthy individuals	TAAD patients:20 Type B aortic dissection (TBAD) patients:15 Healthy subjects:20	Plasma	AD	Lysophosphatidylcholines and Sphingolipids metabolic pathway
Zhang et al^171^	China	Comparison of metabolites secreted by AA aortic wall and AD aortic wall	AA patients:9 AD patients:9	Aortic tissue	AAD	Galactose metabolic pathway
Ren et al^169^	China	Comparison of peripheral blood metabolites between AD patients and healthy individuals	AD patients:25 Hypertension patients:25 Healthy subjects:25	Peripheral blood	AD	Tryptophan, histidine, and glycerophospholipid metabolic pathway
Yang et al^23^	China	Comparison of plasma metabolites between thoracic aortic aneurysm (TAA), thoracic aortic dissection (TAD) patients and healthy controls.	TAA patients:70 TAD patients:70 Healthy controls:70	Plasma	AAD	Sphingolipid metabolic pathway
Huang et al^168^	China	Identification of plasma lipidomics for patients with aortic dissection	AD patients:35 Healthy subjects:32	Plasma	AD	Lipid metabolic pathway
Wang et al^170^	China	Identification of plasma amino acid profile in patients with AD	CHD patients:11 Acute AD patients:11 Chronic AD patients:11	Plasma	AD	Amino acids metabolic pathway
Jiang et al^198^	China	Comparison of serum oxylipin profiles between acute aortic dissection patients and healthy individuals	TAAD patients:10 TBAD patients:10 Healthy controls:10	Serum	AD	Arachidonic acid metabolism pathway
Cibrowski et al^166^	Spain	Comparison of plasma metabolites between patients with aneurysms and healthy subjects	AA Patients: 30 Healthy subjects: 11	Plasma	AA	Sphingolipids, lysophospholipids, and cholesterol metabolic pathway

## Abbreviations

AAD: Aortic aneurysm and dissection
CHD: coronary heart disease
ECM: extracellular matrix
LMWMs: low-molecular-weight-metabolites
NMR: Nuclear magnetic resonance
MS: mass spectrometry
GC-MS: gas chromatography-mass spectrometry
LC-MS: liquid chromatography-mass spectrometry
CE-MS: capillary electrophoresis-mass spectrometry
IMS-MS: ion mobility spectrometrymass spectrometry
CVD: cardiovascular disease
AMI: acute myocardial infarction
PE: pulmonary embolism
AAA: abdominal aortic aneurysms
DAO: diamine oxidase
AF: atrial fibrillation
CR: Caloric restriction
TMAO: trimethylamine N-oxide
SCFA: short-chain fatty acid
BA: bile acid
LPS: lipopolysaccharide
DNA: Deoxyribonucleic Acid
RNA: Reibonucleic Acid
VSMC: vascular smooth muscle cell
UDCA: ursodeoxycholic acid
TUDCA: tauroursodeoxycholic acid
BA: bile acid
SCFA: short-chain fatty acid
ER: endoplasmic reticulum
NRF2: nuclear factor erythroid 2
TMA: trimethylamine
FMO3: flavin lpscontaining monooxygenase 3
LPS: lipopolysaccharide
ROS: reactive oxygen species
IL-6: interleukin-6
IL-1β: interleukin-1β
pro-BDNF: brain-derived neurotrophic factor precursor
ATP: adenosine triphosphate
MAT: methionine adenosyltransferase
SAM: s-adenosylmethionine
SAH: S adenosyl L homocysteine
MS: methionine synthetase
SAHH: S adeno sylhom ocysteine hydrolase
Tfh: follicular helper T cells
Th17: T helper cell 17
NADPH: nicotinamide adenine dinucleotide phosphate
β2GP1: β2-Glycoprotein 1
MMP2: matrix metalloproteinase 2
MMP9: matrix metalloproteinase 9
MCP-1: monocyte chemoattractant protein 1
Arg: arginase
Cys: cysteine
Ang 2: angiopoietin 2
iNOS: inducible nitric oxide synthase
Sirt1: Sirtuin1
ARG1: arginase 1
ARG2: arginase 2
NO: nitric oxide
ADMA: asymmetric dimethylarginine
eNOS: endothelial nitric oxide synthase
EC: endothelial cell
NOX: NAD(P)H oxidase
3-HK: 3-hydroxykynurenine
Kyn: kynurenine
QA: quinolinic acid
3-HAA: 3-hydroxyaminobranoic acid
TDO: tryptophan-2,3-dioxygenase
IDO: indoleamine 2, 3-dioxygenase
AA: arachidonic acid
DHA: docosahexaenoic acid
EPA: eicosapentaenoic acid
Treg: regulatory T cells
GLA: gamma linolenic acid
DGLA: dihomo gamma linolenic acid
PGs: prostaglandins
COX: cyclooxygenase
VCAM-1: vascular cell adhesion molecule 1
ICAM-1: intercellular adhesion factor 1
MCP-1: monocyte chemoattractant protein 1
TCA cycle: tricarboxylic acid cycle
HSD: high salt diet
α-KG: alpha‑ketoglutarate
ACO2: aconitase-2
Acetyl-CoA: acetyl coenzyme A
OGDH: oxoglutarate dehydrogenase
ETC: electron transport chain
NR1D1: NR subfamily 1 group D member 1
CREB: cyclic adenosine monophosphate-responsive element-binding protein 1
6βOHT: 6βhydroxytestosterone
UA: Uric acid
TAD: thoracic aortic dissection
TAA: thoracic aortic aneurysm
Aza: Azathioprine
JNK: c-Jun-terminal-N-kinase
HDL: High density lipoprotein
PCSK9: proprotein convertase subtilisin-kexin type 9
LDL: low-density lipoprotein cholesterol
